# Assessment of Knowledge of Sexual Reproductive Health Among Female University Students in Jordan

**DOI:** 10.7759/cureus.53386

**Published:** 2024-02-01

**Authors:** Mais Alkhalili, Yamamah Al-Hmaid, Khalid Kheirallah, Lama Mehaisen

**Affiliations:** 1 Department of Public Health and Community Medicine, Al-Balqa Applied University, Al-Salt, JOR; 2 Department of Public Health, Jordan University of Science and Technology, Amman, JOR; 3 Department of Obstetrics and Gynaecology, Al-Balqa Applied University, Al-Salt, JOR

**Keywords:** jordan, female university students, sexual reproductive health, knowledge, assessment

## Abstract

Background: Sexual reproductive health (SRH) is an important aspect of human life, especially in the younger age groups. Young adults are the most vulnerable group to SRH consequences, as they have limited access to its information and services. This is one of the earliest studies conducted to examine the knowledge of SRH among female university students in Jordan. The aim of this study is to assess the knowledge of SRH among female Jordanian university students and to determine the social and individual factors that may affect this knowledge.

Methodology: A cross-sectional design was used, and a convenient sample consisting of 427 female university students was recruited from the University of Jordan. The inclusion criteria were female unmarried students aged 18-25 years old, while the exclusion criteria were married, divorced, or engaged female students. A valid and reliable self-administered questionnaire was used to assess the student’s knowledge of SRH. Data were collected between February 21 and March 20, 2022. IBM SPSS Statistics for Windows, Version 23.0 (Released 2015; IBM Corp., Armonk, New York, United States) was used for data analysis, and an independent sample t-test was used to investigate mean differences in the SRH score based on demographic characteristics.

Results: Overall, it was found that only 26.2% of all the participants had adequate knowledge of SRH. Additionally, they have inadequate knowledge regarding the different aspects of SRH such as premarital, vaccination, menstruation, contraception, and sexually transmitted diseases (STDs), except for the section on folic acid, which demonstrated adequate knowledge. Furthermore, the participants’ scores of total SRH knowledge were significantly different based on their original place of living, current residency, educational level, religion, and university faculty.

Conclusion: Due to the inadequate level of knowledge, this study highlights the need to establish educational and awareness programs concerning SRH and to incorporate this subject into the university and school curricula.

## Introduction

Sexual and reproductive health (SRH) is defined by WHO as “a state of physical, emotional, mental, and social well-being concerning sexuality, and not merely the absence of disease, function, or infirmity" [1,2]. It encompasses numerous topics such as sexually transmitted diseases (STDs), vaccination, family planning, nutritional supplements, and pregnancy [1]. WHO declared that SRH issues account for over 20% of the worldwide burden of women’s morbidity [3], and over 1,000 maternal deaths are due to pregnancy or delivery, with 90% of these deaths occurring in Africa and Asia arising from complications such as heavy bleeding, infections, and obstructed labor [3].

Proper SRH propagation is lacking in many developing countries [4]. Several factors have contributed to the emergence of a mismatch of global trends in reproductive health and the situation in developing countries, such as the absence of suitable SRH services, lack of skilled teachers who can transmit sensitive information, and stigma in society [4]. In the Middle East and North Africa (MENA) region, knowledge in the field of SRH is limited, particularly in sexuality issues [4,5].

In Jordan, the percentage of young people aged 15-24 years, during which puberty and sexual maturity take place, comprises 21% of the population [6,3]. The youth group goes through many biological, physical, and psychosocial changes [7]. This period is crucial for social growth and the acquisition of healthy behaviors [2]. For this age group, information on reproductive health is scanty and limited, even among university students [6]. Due to sociocultural constraints, the youth faces many challenges in obtaining adequate knowledge and meeting their necessary needs regarding SRH [1,7].

Also, lack of knowledge may lead to dire consequences such as unintentional pregnancy and unsafe abortion, especially in developing countries, that will negatively affect child and maternal mortality [8,9]. Equally important, young females encounter different issues such as early pregnancy, unsafe abortion, sexual violence, and underuse of contraception [7,10]. Young people aged 18-24 are the most vulnerable group for unintended pregnancy [11]. In addition, they have lower rates of usage of healthcare services compared with other age groups [11].

Jordanian youth have tremendous needs for SRH information and services which have not been satisfied owing to various causes. Reproductive health services are both unappealing and insufficient to the youth [12], their parents are unprepared to discuss sexually related topics due to a lack of knowledge and the culture of shame in society, and healthcare providers do not fulfill the requirements of youth and do not give adequate or valid information [12,5].

According to the Ministry of Health in Jordan, barely 1% of teenagers receive primary healthcare, and the SRH services offered to them are frequently of low quality [12,5]. SRH services are mostly focused and delivered primarily to females, particularly in the areas of contraception, antenatal care, and the preservation of secure maternity [13].

SRH education programs at the university level have been shown in the literature to be a cost-effective and excellent means of fostering healthy habits [6]. SRH education enables women to comprehend their responsibilities and rights [4]. SRH is seldom covered in the Arab education curriculum, and it is usually disregarded by instructors who encounter difficulties in dealing with this issue [6].

While various studies have revealed the shortage of knowledge regarding SRH among Jordanians, and hence the restricted usage of its services [6,14], this study is one of the earliest to examine the knowledge of female university students on the subject.

The importance of this study lies in the ability to make a change in the university curriculum, and in the methods of health education at the level of SRH [1,6]. Since university students are in the pre-marital period, it is very important to instruct them on SRH [6]. Thus, this study aimed to assess the knowledge of SRH among female Jordanian university students, and to determine the social and individual factors that may affect this knowledge. The target group was this category of women who are still not married and have supposedly not been engaged in any sexual experience.

## Materials and methods

This cross-sectional study was conducted to assess the level of knowledge about SRH among female students at the University of Jordan, Amman, Jordan, and to investigate the mean differences in SRH knowledge scores based on demographic data. The University of Jordan is a public university and females constitute approximately 66% of the students at the university, with a total of almost 36,300 female students currently studying there. The study was approved by the Jordanian Ministry of Health’s Institutional Review Board (approval number: Moh/REC/2021/245).

Female, unmarried students aged 18-25 years were included in the study. Married, divorced, or engaged female students were excluded because they may have already acquired knowledge from their marital experience. Participants were reassured that participation was voluntary. They were also reassured that their replies would be treated confidentially and would not be shared with anybody. Informed consent was included in the questionnaire for the participants and they were informed that their agreement to complete the questionnaire constituted their consent to participate.

Sample size

Participants were chosen by non-probability convenience sampling. The sample size was calculated using Slovin’s formula [15]:

 n= N/(1+ N*€2)

Where N is the population size, € is the margin of error, which equals 0.05 in a 95% confidence interval.

The sample size was 396 participants.

Data collection

Data were collected about participants’ demographics, premarital tests, vaccination, menstruation, pregnancy and its symptoms, contraception, vitamins, and STDs. Data were gathered utilizing a self-administered questionnaire between February 21 and March 20, 2022. The questionnaires were collected in soft copies using online Google Forms (Google LLC, Mountain View, California, United States). Participants in the university who fulfilled the inclusion criteria were asked to participate in the study by sending them a link via phone messages or e-mails to fill out the questionnaire.

Tool

The research tool was adapted from previous related studies [1,2]. Cross-checking and verification were done by specialists to ensure the study instrument's content validity. Following that, changes were made in accordance with the advice and recommendations. A pilot research sample of 38 students (10% of the overall sample size) was enrolled to assess the questionnaire's feasibility and appropriateness, as well as to estimate the time required to complete it. The questionnaire was modified in response to any input received during the pilot trial. The questionnaire took around 10 minutes to complete. The tool was deemed to be practical, obvious, and appropriate after no adjustments were made based on the outcome. Eligible participants were then asked to fill out the validated, self-administered questionnaire.

The validated questionnaire had seven sections: (i) Socio-demographic section included questions about age, nationality, educational level of the participant, the place of residence, academic level of parents, religion, people who they lived with, and work; (ii) Premarital tests section comprised questions about blood group compatibility between couples, and some premarital screening tests like thalassemia and diabetes; (iii) Vaccines section involved questions about human papillomavirus (HPV), tetanus, hepatitis B virus, and measles-mumps-rubella (MMR) viruses; (iv) Menstruation section comprised questions about duration of menses, ovulation, amenorrhea, and dysmenorrhea, in addition to questions about pregnancy detection, and timing of pregnancy; (v) Knowledge about contraception included questions about methods of birth control such as combined oral contraceptive pills (COCPs), progesterone-only pills, and intrauterine devices (IUD); (vi) STD section included questions about the mode of transmission, symptoms, and HIV-related questions; and (vii) Vitamins section tested the participants’ knowledge with regard to folic acid. Afterward, the reliability was assumed based on the Kuder-Richardson Formula 21 (KR-21) due to dichotomy answers and found to be 0.77 for all the questionnaire’s items.

Scoring system

Each right answer received 1 point, while wrong answers and "I don't know" responses received 0 points. The first part (The sociodemographic section) was left out of the scoring system because it contained qualitative data. In the second section (the premarital tests section), the total score for correct responses was calculated to be 5; in the third section (the vaccines section), the total score was 8; in the fourth section (the menstrual cycle and pregnancy section), the total score was 9; in the fifth section (the contraception section), the total score was 27; in the sixth section (STD), the total score was 15; and finally in the seventh section (vitamins), the total score was 2. So, the total score of accurate answers for the entire questionnaire was 66. By aggregating the results from the seven sections, a new index was created to measure total knowledge. The mean±SD for each section score was calculated, and then the total score for each section was divided into inadequate and adequate knowledge levels based on their corresponding 75th percentile.

Data analysis

IBM SPSS Statistics for Windows, Version 23.0 (Released 2015; IBM Corp., Armonk, New York, United States) was used to conduct the statistical analysis. The sample and primary study variables were initially described using descriptive statistics (mean, SD, and frequencies). An independent sample t-test was used to investigate mean differences in SRH scores based on demographic characteristics. The results were considered statistically significant if P value ≤ 0.05.

## Results

This study included 427 female university students. The majority of them were from medical colleges (n=253, 59.3%), and had a bachelor’s degree in education (n=409, 95.8%) with a mean age of 21.49 ±1.91 years. In terms of their living conditions, most of them lived with their families or friends (n=398, 93.2%), and resided in the capital of Jordan, Amman (n=292, 68.4%). A detailed summary of the study participants’ characteristics is shown in Table 1.

**Table 1:** Characteristics of the study participants (N=427)

Characteristics	Frequency	Percentage
The original place of residence		
Amman	292	68.4
Other regions	135	31.6
Staying with		
Alone	29	6.8
Family or friends	398	93.2
Nationality		
Jordanian	369	86.4
Non-Jordanian	58	13.6
Educational level		
Bachelor degree	409	95.8
Higher degrees	18	4.2
Religion		
Muslim	413	96.7
Christian	14	3.3
University specialty		
Medical College	253	59.3
Other specialties	174	40.7
Work status		
Only student	345	80.8
Student and working	82	19.2
Mother's education		
High school or less	134	31.4
University	293	68.6
Father's education		
High school or less	112	26.2
University	315	73.3
Age (years), mean (SD)	21.49 (1.91)	

The SRH questionnaire was used to measure unmarried female university students’ knowledge regarding six SRH sections, namely premarital, vaccination, menstrual cycle, contraception, STD, and folic acid sections, with a total of 66 questions. Table 2 summarizes the mean±SD for each total section score. The total score for each section was divided into inadequate and adequate knowledge levels based on its corresponding 75th percentiles.

**Table 2:** Overall sexual and reproductive knowledge presented according to the different sections STD: Sexual Transmitted Disease

Sections of the questionnaire	Mean (SD)	Level of knowledge based on 75th percentile		
		Inadequate, n (%)	Adequate, n (%)	
Premarital test knowledge	2.77 (1.20)	307 (71.9%)	120 (28.1%)	
Menstruation knowledge	6.56 (2.46)	236 (55.3%)	191 (44.7%)	
STD knowledge	7.96 (3.56)	293 (68.6%)	134 (31.4%)	
Folic acid knowledge	0.63 (0.53)	169 (39.6%)	258 (60.4%)	
Vaccination knowledge	3.67 (2.02)	261 (61.1%)	166 (38.9%)	
Contraception knowledge	11.97 (5.94)	314 (73.5%)	113 (26.5%)	
Overall knowledge	33.57 (12.87)	315 (73.8%)	112 (26.2%)	

The mean knowledge score was calculated for all sections and found to be 33.57±12.87. Then the 75th percentile was computed and found to equal to 43.0. Based on this cut-off point, the total knowledge score was categorized. A total of 315 (73.8%) participants had inadequate knowledge and 112 (26.2%) had an adequate knowledge level of SRH. This is summarized in Table 2.

Furthermore, the participants’ total scores were significantly different based on original place of living, current residency, educational level, religion, and university education as summarized in Table 3. In terms of the original place of living, students living in Amman had significantly higher total SRH knowledge mean score (35.22, SD=11.68) than students living in other regions (29.99, SD=14.52) (t(425)=3.40, p<0.001). Students living with family or friends had significantly higher total SRH knowledge mean score (34.09, SD=12.50) than students living alone (26.45, SD=15.76) (t(425)=3.12, p=0.002). Similarly, students who had a bachelor’s degree had significantly higher total SRH knowledge mean score (34.08, SD=12.33) than students who had a higher degree (21.94, SD=18.75) (t(425)=3.40,p<0.001). Muslim female students had significantly higher total SRH knowledge mean score (33.91, SD=12.50) than Christian female students (23.57, SD=19.31) (t(425)=3.00, p= 0.003). Also, medical students had significantly higher total SRH knowledge mean score (36.41, SD=10.90) than students in other specialties (29.44, SD=14.35) (t(425)=5.70, p<0.001). However, the means of nationality (p=0.827), work (p=0.409), mother's education (p=0.620), and father's education (p=0.592) were not found to be significantly related to total SRH knowledge mean score.

**Table 3:** Factors affecting students’ total sexual reproductive health knowledge score

Characteristics	Mean	p-value
Place of living		<0.001
Amman	35.22	
Other regions	29.99	
Staying with		0.002
Alone	26.45	
Family, friends	34.09	
Nationality		0.827
Jordanian	33.5	
Non-Jordanian	33.91	
Educational level		<0.001
Bachelor degree	34.08	
Higher degrees	21.94	
Religion		0.003
Muslim	33.91	
Christian	23.57	
University specialty		<0.001
Medical College	36.41	
Other specialty	29.44	
Work status		0.409
Only student	33.82	
Student and working	32.51	
Mother's education		0.620
High school or less	33.11	
University	33.78	
Father's education		0.592
High school or less	33.01	
University	33.77	

## Discussion

This study aimed to determine the level of SRH knowledge among single, unmarried female students at the University of Jordan. The participants' total level of knowledge was discovered to be 26.2%. This can be explained by the lack of awareness and educational programs in schools or universities, as well as the lack of communication with parents, as most of them are hesitant to discuss these matters with their children, in addition to the limited availability of access to this information, especially through social media, since this topic is considered taboo in society [9].

The findings were consistent with research on young individuals in Lebanon, where the knowledge was 8.8% as per Hamdanieh et al., who conducted a cross-sectional study of 491 single, unmarried women residing in Lebanon, aged 17-55 years [1]. Furthermore, a literature review of female university students in the Middle East revealed a lack of SRH knowledge [6]. This can be attributed to the shortage of sexual health education courses in universities, in addition to the lack of media coverage of sexual and reproductive health issues [14].

Meanwhile, a study by Santos et al. mentioned that the level of knowledge was moderate (57%) in 1946 college students in Portugal with a mean age of 21 years [15]. Moreover, a cross-sectional study in Ethiopia carried out on 419 university students demonstrated adequate knowledge of participants (59.6%) regarding SRH [16]. This brings attention to young people's SRH situation, concerns, and obstacles in Jordan, and this study may assist in setting suitable plans, initiatives, and interventions for youth.

Besides, the result of premarital knowledge was found to be inadequate by the current study. The reason behind this finding may be explained by the lack of educational programs in schools and universities that raise awareness about hereditary diseases, especially in societies that have high rates of consanguinity. It’s also worth mentioning the obligation of the public health law in Jordan for couples to test for thalassemia before marriage to detect thalassemia traits in siblings [17]. On the other hand, another study carried out at Amman Arab University showed an adequate level of knowledge regarding premarital examination [18]. Similarly, adequate knowledge was also seen in a study in Saudi Arabia [19]. Consequently, authoritative institutions such as healthcare staff who are sufficiently knowledgeable and trained to convey appropriate information must address this issue, assist couples in identifying possible health problems, and inform them about the likelihood of passing these hazards on to their offspring [20].

Knowledge of HPV vaccine administration was found to be inadequate by the current study. This can be because of the failure of the media to raise awareness and spread the importance of receiving this vaccine, in addition to the lack of care of health personnel to talk about it as it is not included in the Jordanian national vaccination program. This result was congruent with a cross-sectional study conducted among United States college students aged 18-25 years to determine their knowledge and attitude toward HPV vaccination. The participants in the study had limited knowledge, and those who had been vaccinated had much greater knowledge than their non-vaccinated peers [21]. However, the level of knowledge concerning HPV vaccination was moderate (44.5%) in a cross-sectional study that was conducted in Turkey by Rathfisch et al. on 605 university students to measure the level of knowledge and behavioral intentions regarding HPV [22]. Moreover, in a cross-sectional study conducted in Jordan among female university students to investigate readiness and attitude about the HPV vaccination and awareness of the virus, 62.7% of participants had known of HPV before the survey, of whom 48.7% were aware that HPV vaccinations were accessible in clinics [23]. Young people in Jordan should be given correct information about HPV through community-based teaching programs and medical services to draw attention and establish healthy habits. However, since the vaccine is not present in the national vaccination program, greater effort must be made to familiarize people with it, as it is only available in the private sector.

Moreover, the outcomes of menstrual knowledge were found to be insufficient. More than half of the students did not distinguish between an ordinary menstrual cycle (28-35 days) and a menstruation period (less than eight days). This might be because obtaining information and seeking support on a topic shrouded in secrecy is challenging. This result was supported by Arshad et al., who revealed an insufficient understanding of menstruation among women in Pakistan which necessitates engaging gynecological health classes at the school level for young girls to address this knowledge gap and eliminate incorrect concepts [24]. On the other hand, in a questionnaire-based survey conducted among 2572 female university students in Hungary, Romania, and Serbia, the knowledge of the participants regarding the relevance of menstruation in sexual health was found to be adequate [25]. Encouraging healthcare professionals and mothers to educate young girls in the general community about monthly abnormalities and how they affect their reproductive health is a primary concern to help bridge the knowledge gap. Moreover, it’s essential for the Ministry of Education to include this material in the school curriculum, especially for high school students, and to organize awareness programs for them to discuss this subject scientifically.

Furthermore, the results for knowledge of contraception reflected an inadequate level of knowledge. This may be attributed to the lack of counseling sessions by health personnel for women of childbearing age when they visit the maternity clinics. This result is supported by Hamdanieh's study, which included 491 single unmarried females aged 17-55 years and revealed a low level of knowledge of contraception (13.5%) [1]. Also, a literature review of studies related to contraception that involved young women aged 11-24 years in developing countries (sub-Saharan Africa and South-East Asia) detected a low level of knowledge [26]. On the other hand, a study carried out among 758 college students in India to find out the level of knowledge regarding contraception revealed a moderate level of knowledge (60.1%) [27]. In Jordan, almost all ever-married women (99.1%) are familiar with some method of contraception, and they have heard of approximately eight different methods among which the intrauterine device (IUD) is the most popular [28]. Empowering health providers to give counseling sessions to women of reproductive age and setting policies that enable the staff to provide such a service is essential to improving knowledge and use of contraceptive methods.

Furthermore, the level of knowledge about STDs was inadequate. An obvious reason is the fact that schools and institutions do not adequately teach students about STDs. The current finding is relatively consistent with the study of Nigussie et al. in Ethiopia, which revealed an inadequate level of knowledge [29]. On the contrary, adequate knowledge of STDs was demonstrated in Greece [30]. This emphasizes the need to establish more effective sexual education programs for adolescents in the university curriculum in Jordan. Further, it is strongly advised to use social media to improve information, education, and communication on this issue.

Concerning folic acid, the results proved to be adequate. This result may be due to the increase in access to reproductive health services for women as they are more aware of its importance in preventing neural tube defects. Also, good knowledge can be connected to reliable sources of information such as health professionals and teachers [22]. Our findings were also congruent with a cross-sectional study that was conducted among university students in Ukraine that aimed to explore the level of knowledge of folic acid, which demonstrated an adequate level of knowledge [31]. In a cross-sectional study by Mroczek et al. among female students of reproductive age, the findings revealed an inadequate understanding of folic acid supplementation [32]. In Jordan, maternal health clinics should continue providing women with appropriate information regarding the uses of folic acid and its effects.

Study strengths and limitations

The study's greatest strength is that it is one of the earliest studies to assess knowledge of SRH among female university students at the University of Jordan. Also, using a self-administered questionnaire via online Google Forms provided convenience and accessibility, which saves time and money. It also adds an important database to follow up with university students.

One of the limitations is the use of convenience sampling rather than random sampling, which was difficult to implement because students had examinations at the time of data collection and many of them were not residing at the university. Moreover, the sample doesn’t represent all the students in Jordan, as it was taken from only one university. Furthermore, not including males in the study was a limitation because the male partner has an indispensable role in promoting SRH; however, we did not include males in our study because we expected to have a high non-response rate.

Recommendations of the study

Stakeholders in the Ministry of Higher Education, in collaboration with the Ministry of Health, should adjust university curricula by incorporating SRH content for students; for example, they can include SRH mandatory courses into the universities’ curricula. Further studies are recommended and the current study can be the bridge for many researchers in this field using other research methods to learn more about this topic. Also, the Ministry of Health can set up educational programs in cooperation with social media and non-governmental organizations for women of childbearing age to give information about SRH and services related to it.

## Conclusions

This study revealed that participants had inadequate knowledge about SRH. Additionally, they have inadequate knowledge regarding certain sections of SRH such as premarital tests, vaccination, menstruation, contraception, and STDs. Adequate knowledge was seen in the section on folic acid. Furthermore, the association of participants’ SRH knowledge with their original place of living, current residency, educational level, religion, and university education was statistically significant. This study highlights the need to establish educational and awareness programs on SRH and to incorporate this subject into university and school curricula.
